# Building Phylogenetic Trees From Genome Sequences With kSNP4

**DOI:** 10.1093/molbev/msad235

**Published:** 2023-11-09

**Authors:** Barry G Hall, Jeremiah Nisbet

**Affiliations:** Bellingham Research Institute, Portland, OR, USA; Bellingham Research Institute, Portland, OR, USA

**Keywords:** sequences, kSNP4, SNPs

## Abstract

Performing phylogenetic analysis with genome sequences maximizes the information used to estimate phylogenies and the resolution of closely related taxa. The use of single-nucleotide polymorphisms (SNPs) permits estimating trees without genome alignments and permits the use of data sets of hundreds of microbial genomes. kSNP4 is a program that identifies SNPs without using a reference genome, estimates parsimony, maximum likelihood, and neighbor-joining trees, and is able to annotate the discovered SNPs. kSNP4 is a command-line program that does not require any additional programs or dependencies to install or use. kSNP4 does not require any programming experience or bioinformatics experience to install and use. It is suitable for use by students through senior investigators. It includes a detailed user guide that explains all of the many features of kSNP4. In this study, we provide a detailed step-by-step protocol for downloading, installing, and using kSNP4 to build phylogenetic trees from genome sequences.

## Introduction

Molecular phylogenetic analysis always requires comparing homologous sites within a set of DNA, RNA, or protein sequences. As genes diverge, they occasionally experience small insertions and deletions, and therefore, the first step in phylogenetic analysis of genes is to identify the homologous sites by *aligning* the gene sequences. For a long time, the development of sophisticated alignment programs was a major focus of effort in the fields of evolution, systematics, and bioinformatics. Those programs could align a few hundred sequences of a few thousand nucleotides each.

In the 1990s, new technologies permitted sequencing not just individual genes but entire microbial genomes consisting of 5 to 6 million nucleotides. It was initially assumed that aligning genome sequences would just require improved alignment algorithms and bigger, faster computers. However, that turned out not to be the case. Programs that were capable of aligning >30 genomes were not available, and aligning even 25 genomes required over 70 h (Gardner and Hall 2013); thus, it appeared that it would not be possible to do a genome-based phylogenetic analysis of any but the smallest data sets.

Gardner and Slezak (2010) changed molecular phylogenetics forever by eliminating the need to align sequences in order to identify homologous sites. She reasoned that short sequences, called *kmers*, would be homologous if they were identical at all but the central site in odd-length kmers. The kmers had to be long enough that they would not occur by chance alone. That central site in the homologous kmers constituted a single-nucleotide polymorphism, or SNP, and the homologous SNP sites could be directly compared with estimate a phylogeny. kSNP2 followed in 2013 and kSNP3 followed in 2015 (Gardner and Hall 2013; Gardner et al. 2015).

kSNP4 identifies SNPs and does phylogenetic analysis without genome alignment or the use of reference genomes. It is primarily used for the analysis of viral, bacterial, and fungal genomes. The input data are genome sequences in FASTA format. They may be complete genome sequences, genome assemblies, or raw reads. They estimate phylogenetic trees based on 3 methods: parsimony, neighbor ioining (NJ), and maximum likelihood (ML). Hall (2015) showed that for SNP-based phylogenies, in which there are no invariant sites, parsimony is the most accurate method of phylogeny reconstruction. If one or more annotated genomes are included in the data set, kSNP4 can automatically download the annotations and annotate the SNPs. kSNP has been widely used and, together, has been cited over 700 times.

In October 2022, Hall and Jeremiah Nisbet released kSNP4.0. kSNP4 restored the annotation function that was lost when NCBI discontinued the use of gi numbers and is more than twice as fast as was kSNP3. kSNP4 is freely available for Linux and Mac OS operating systems at https://sourceforge.net/projects/ksnp/files/. The most recent version, kSNP4.1, is capable of analyzing 500 *Escherichia coli* genomes in slightly over 9 h on a desktop Linux computer. The time required is a function of the cube of the number of genomes (supplementary fig. S1, Supplementary material online). A complete and detailed user guide is included. kSNP4 requires no programming skills, no bioinformatics experience, and no additional programs (dependencies). It is suitable for everyone from students to senior investigators. For those who are not familiar with the command-line interface, a separate guide to using that interface is included in the kSNP4 documentation.

## Why Base Phylogenetic Trees on Genome Sequences?

The most common application for kSNP has been for the analysis of prokaryotic, eukaryotic, and viral microbial genomes, where phylogenetic analysis is important to lineage inference, outbreak tracking, microbial epidemiology, and phylogenomics. While the major phylogenetic methods (parsimony, NJ, and ML) have been well established for decades, the methods for distinguishing taxa from each other have undergone rapid changes. In this context, taxa are the individual microbial isolates, and the method of distinguishing isolates from each other is called “strain typing.” Sequence-based typing methods have evolved as the sequencing technology evolved and became faster and less expensive. Whole-genome sequences provide the highest possible resolution in strain typing.

## Valuable Companion Programs

Whole-genome sequences provide the ultimate strain typing resolution—indeed the resolution can be too good. The key question in tracing the outbreaks of pathogens is whether 2 isolates are of the same strain. Isolates of the same strain are unlikely to have identical genome sequences. A recent study (Ankrum and Hall 2017) showed that isolates of the same *Staphylococcus aureus* strain could have up to 70 SNP differences. The pairwise SNP differences among genomes were determined using the companion program *kSNPdist*, which is freely available at https://sourceforge.net/projects/ksnp/files/.

Some microbial phenotypes such as virulence, are either difficult or very time-consuming to determine. When following outbreaks, it can be valuable to use genomic sequences to predict the phenotypes of new isolates. The companion program *PPFS2* (Hall 2014) identifies the SNPs that are causally associated with phenotypes. PPFS2 for Linux and Mac OS operating systems is also freely available at https://sourceforge.net/projects/ppfs/ and includes complete documentation.

Neither companion program is required for using kSNP4.

## Why Use kSNP4 for SNP Analyses?

kSNP4 both identifies SNPs in the data set and estimates phylogenetic trees from those SNPs. There are numerous other programs that either identify SNPs or estimate trees from SNPs, but we discuss only those that do both.

kSNP4 does not require reference genomes, does not require bioinformatics experience to install or use, and does not require dependencies that are not included in the kSNP4 package, but it is capable of annotating SNPs. It includes a detailed user guide. I am unaware of other programs or pipelines that meet all of those criteria.

There are several other programs available that use SNPs to estimate phylogenetic trees. I have excluded programs that find only SNPs but do not estimate phylogenetic trees. Likewise, I have excluded programs that require SNP data files as the input.

RealPhy (Bertels et al. 2014), SNVPhyl (Petkau et al. 2017), Lyve-SET (Katz et al. 2017), and PhaME (Shakya et al. 2020) all require a reference genome to identify SNPs. This is disadvantageous because the only SNPs that can be identified in the data set genomes are those that are present in the reference genome. SNphylo (Lee et al. 2014) requires considerable programming and computer skills to install and use. The website no longer exists and there is no manual. Similarly, SNVPhyl, RealPhy, and Lyve-SET require considerable experience in bioinformatics to install and use. PhaMe must be installed from a source code, which requiring significant bioinformatics experience, and estimates trees based only on the core SNPs.

## Step-by-Step Protocol

Here, we provide a step-by-step protocol for building phylogenetic trees from genome sequences with kSNP4. We use courier font to indicate what should be typed into the Terminal.

### Step 1: Download and Install kSNP4

kSNP4 is a command-line program and is accessed through the Terminal application. Users unfamiliar with command-line programs should review “A command-line primer” that is included in the documentation folder that is part of the kSNP4 package. The primer discusses the installation and use of command-line programs.

#### Step 1a: Download kSNP4

Download either the *kSNP4.1 Mac Package* zip file or the *kSNP4.1 Linux Package* zip file from SourceForge https://sourceforge.net/projects/ksnp/files/. When the zip archives are extracted, each contains a *kSNP4.1pkg* directory (folder) and a documentation directory that includes the user guide and the command-line primer.

#### Step 1b: Install kSNP4

In Terminal, navigate to the kSNP4 package directory that was extracted from the downloaded zip file. To install the kSNP4.1pkg directory within it into the /usr/local directory, enter: sudo cp -R kSNP4.1p

kg /usr/local. When asked for a password, enter the administrator’s password for the current user. (If you are not the administrator, find the person who is and get them to authenticate for you.)

To check whether the package was actually moved to /usr/local enter cd /usr/local, enter ls to list the contents of /usr/local. You should see kSNP4.1pkg listed. If you do not see it listed, try again or get help from your computer’s administrator.

#### Step 1c: Set the PATH Variable

For you to be able to use kSNP4, the operating system needs to be able to find all of the programs in the kSNP4.1pkg when you type kSNP4 on the command line. To do this, you must set the PATH environmental variable to include the path of the kSNP4.1pkg installation (/usr/local/kSNP4.1pkg). A path is just a description of the nesting of directories starting with the root directory (referenced as just /), with the directory names separated by /. The path /usr/local means the local directory that is in the usr directory that is at the root.

To add paths you must modify a file that sets environmental variables. The file is found in your home directory. Under the bash shell, depending on your OS and its age, that file is called.bash_profile or .bashrc, and it is found in your home directory. Under the zsh shell (the default shell in some recent versions of both Mac OS and Linux operating systems), the file that holds environmental variables is called .zprofile, which is also found in your home directory. *In both cases, the file is invisible because its name begins with a dot*.

In your *home* directory in *Linux*, type control-H, or under the *View* menu, choose *Show hidden files*. In *Mac OS*, type Command-shift-period. You should now see the hidden files. Open the hidden file with the name from above in a text editor, then *at the end of the file*, type (or paste in) the following line *exactly as given here,* i.e. with *no spaces around the = sign*: export PATH=“/usr/local/kSNP4.1pkg:$PATH”

The best way to determine whether you have installed kSNP4 correctly is to download the Examples.zip file from https://sourceforge.net/projects/ksnp/files/ and to use those examples in the following steps. The Examples folder includes 2 folders: Example1 and Example2. Each includes a directory of genomes.

#### Step 1d: Memory Management

kSNP4 can use a lot of RAM (memory) if the data set includes hundreds of genomes. The solution is to permit using some hard disk space as memory. The Mac OS manages memory automatically, but for Linux OS, we recommend setting up a swapfile equal to twice the installed RAM. SWAP is a partition of disk space that can be used as virtual memory when real memory starts to run out. This site https://www.ubuntupit.com/how-to-add-and-configure-swap-space-on-ubuntu-linux/ shows you how to increase that space.

### Step 2: Prepare the Input File

Data for estimating a tree consists of a set of genome sequences in Fasta format. The sequences can be a mixture of complete genomes, genome assemblies, and raw reads. All of the sequences should be in a folder (directory) somewhere on your hard drive but NOT in the kSNP4pkg directory. If you do not have one already, we suggest you put a folder in your home directory named genomes. Once created, this directory name should not be modified. Wherever you put the genome sequences, it is important not to move them after you create the kSNP4 input file, otherwise, kSNP4 will not be able to find them until you create a new input file.

#### Step 2a: File Names

It is important that the input files are correctly named. Depending on your operating system, file names that violate the rules below may cause horrible results—including greatly inflating the number of SNPs, or failing to annotate the SNPs correctly. kSNP4 may appear to have run properly, and unless you have had considerable experience with similar data sets, you may not recognize the fact that errors have occurred. To avoid such a situation, kSNP4 checks the input file for file names that violate the rules and aborts if incorrect names are detected.

A file name consists of 2 parts: a file ID and an extension. The extension is separated from the file ID by a dot. For the file named *Escherichia_coli_042.fasta*, the file ID is Escherichia_coli_042 and the extension is fasta.

The rules for naming files are as follows:

- The file name cannot include more than 1 dot (.); i.e. only the dot separating the file ID from the extension is allowed. Escherichia_coli_042.fasta is legal; Escherichia_coli_0.42.fasta is not. (It is legal for your operating system, but it is not legal for kSNP4.)
- The file name may not contain any spaces. Escherichia_coli_042.fasta is legal; Escherichia coli 042.fasta is not.
- The file name can contain only the characters A to Z, a to z, 0 to 9, - (the dash or minus character), and _ (the underscore character). Characters such as *, (, ), and > are illegal. When necessary for readability, replace spaces, colons, etc. with the underscore character or use internal capitalization; for instance, Ecoli_O157H7 instead of Ecoli O157:H7.

If kSNP4 finds a file with an illegal character, it terminates the run and writes a file named NameErrors.txt. If you examine the contents, NameErrors.txt reminds you of the naming rules and writes a list of the files that contain naming errors. You will need to rename the listed files and start over from this point if you encounter issues with file names, so it is important to be careful at this step.

#### Step 2b: Making the Input File

**Fig. 1.**
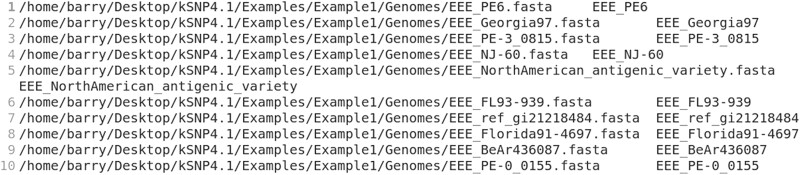
A typical kSNP4 input file.

The program *MakeKSNP4infile*, included in the kSNP4pkg, automatically makes an input file from the genome sequence files in a folder. In Terminal, navigate to the directory that *encloses* the directory containing the fasta genome files and type MakeKSNP4infile -indir myDir -outfile myInfile. myDir is the name of the directory that contains the fasta files and myInfile is the name for the kSNP4 input file that will be written. Substitute the actual names of the directory and the desired name for the kSNP4 input file. For the Example1 data set, in Terminal, navigate to the Example1 folder and type MakeKSNP4infile -indir Genomes -outfile EEvirus.in (equine encephalitis virus). The genome ID for each file is generated from the file name (any extensions, such as .fasta, are dropped). The resulting file named EEvirus.in will appear in the Example1 folder, and it will look like Fig. 1.

Each line consists of a path to a genome sequence file and a genome ID; i.e. EEE_PE-0_0155. The genome ID is the taxon label that will appear on trees written by kSNP4.

**Fig. 2.**
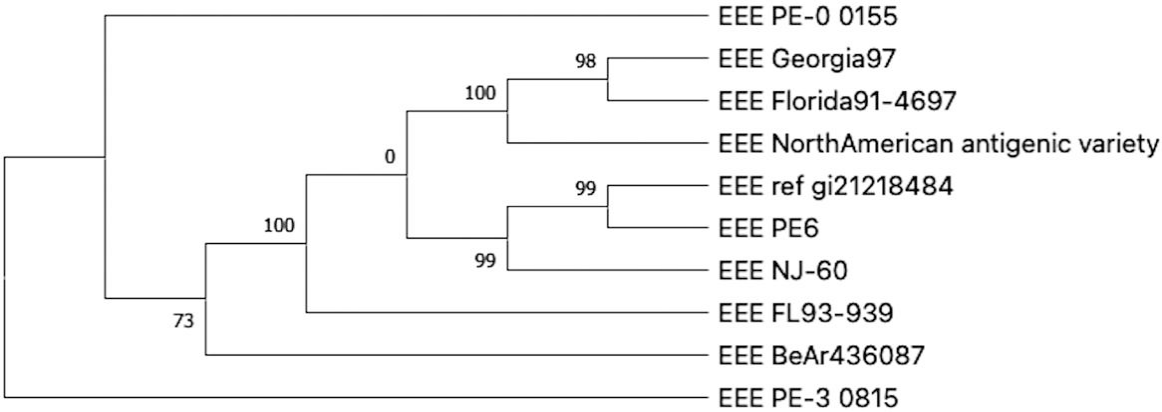
Drawing of a parsimony tree from Example1. The numbers indicate branch support.

Caution! Check to be sure that there are no spaces in the path lines of the kSNP4 input file (for example, see Fig. 2). If there are spaces, it means that a folder has a space in the name. Move the genomes into a folder whose complete path has no spaces and repeat MakeKSNP4infile. Spaces in path names will result in an error like [48632] Failed to execute script “Kchooser4” due to unhandled exception!

### Step 3: Choose a kMER Length

Kmer size, *which must be an odd number*, defines the length of the oligonucleotides (kmers) that kSNP4 identifies in all of the sequences. Oligonucleotides that are identical between different genomes at all but the central base (odd-length kmers are used so that there is a central base) are taken as being homologous, i.e. an SNP locus. If the kmer size is set too low, say to a value of 5 bp, then there will be many kmers that are identical by chance alone within a genome and between genomes, rather than being identical by descent from a common ancestor; i.e. homologous. If the target genomes are short, then the chance of spuriously identical kmers is reduced; whereas long genomes increase the chance of spurious matches. This consideration would seem to favor choosing large values for kmer size. However, if the kmer size is set too high, say 51 bp, then many SNPs will be missed because of sequence variation at multiple sites within the kmer, because an SNP locus is defined by the *conserved* sequence surrounding the central base of the kmer. When there is little base-pair variation, large values reduce the chances of spurious matches, but when there is much variation, large values reduce the sensitivity of SNP detection. The program *Kchooser4* analyzes the specific data set to suggest the optimum kmer size to use. Kchooser4 tries a series of odd-length kmers and seeks a shortest length for which each kmer occurs only once in the median-sized genome (fraction unique kmers, or FUK). Because there may be some duplicated regions in the genome when FUK increases by <0.001, the search for the optimum kmer length terminates.

Kchooser4 also calculates the fraction of core kmers (FCK), those kmers that are present in every genome. A total of 1,000 random optimum length kmers from the shortest genome are tested to determine which are present in all genomes. When FCK is ≥0.1, the topological accuracy of parsimony trees is ≥90% (Hall 2015). FCK is useful in deciding whether the inclusion of genomes from other species in the genus will make tree accuracy unacceptable. Such genomes are often used as outgroup genomes to root a tree.

In Terminal, navigate to the folder (directory) that contains the input file that was made in Step 2. Type Kchooser4 -in infileName. If the input file was named EEvirus.in, type Kchooser4 -in EEvirus.in. The resulting file will be named Kchooser4_EEvirus.report. Open this file in a text editor to see whether, for example, the optimum kmer length is 11.

### Step 4: Run kSNP4

kSNP4 has many options, which are detailed in the user guide. Here, we will discuss only a few of those command-line options. In Terminal, navigate to the directory that contains the input file; in this example, the Example1 folder contains the input file EEvirus.in.

#### Step 4a: The Simplest Run

The command line consists of the command kSNP4, followed by a series of options. The simplest case consists of 3 required options: -in, the infile name, -outdir, the name of the directory where all the output files will be saved, and -k, the kmer length. In Terminal, navigate to the Example1 folder and enter kSNP4 -in EEvirus.in -outdir EEV_Run1 -k 11 |tee Run1Log.txt. Step 3 showed that the optimal kmer length is 11; thus, “-k 11” is added to the command line. The output folder will be named EEV_Run1. The “| tee Run1Log.txt” part of the command line says “save a text file named Run1Log.txt,” while also displaying the same information on the screen. The Log file records everything that appears on the screen during the run and is therefore a valuable record of the conditions during the run.

There will be some screen output as Terminal shows the various steps that kSNP4 is executing. When the run is done, it will show the time used; in this example, on my computer 7 s. The actual time will depend on your computer and the number and size of the genomes (see supplementary fig. S1, Supplementary material online).

The resulting EEV_Run1 folder will contain 20 files, all of which can be read in your favorite text editor. The COUNT_SNPs file shows the number of SNPs in the data set; in this example, 2,626. The SNPs_all_matrix.fasta file shows an “alignment” of the SNPs in FASTA format.

The tree.parsimony.tree file is a Newick file of the default parsimony tree. The parsimony tree is a consensus of up to 100 equally parsimonious trees. The internal node labels show the support for that node as calculated by FastTreeMP (Price et al. 2010), as the number of trees that include the taxa descended from that node. The tree is unrooted.

The user guide discusses the contents and interpretation of each of the output files.

kSNP4 does not draw the tree diagrams with which you are probably familiar. It simply records all the tree information in a file in Newick format. These files can be read by any tree-drawing program such as MEGA (Tamura et al. 2021). Figure 2 shows the Example1 parsimony tree as drawn by MEGA11.

#### Step 4b: Adding Core SNPs

kSNP4 can also estimate a tree just from the core (common to all) SNPs. The command would be kSNP4 -in EEvirus.in -outdir EEV_Run2 -k 11 -core |tee Run2Log.txt.

By adding the -core option, the number of output files was increased to 35 including COUNT_coreSNPs; in this example, 67. The tree file based only on the core SNPs is “tree.core_SNPs.parsimony.tre,” but notice that the tree based on all the SNPs, tree.parsimony.tre, is present in this run. The -core option in the command adds the analysis based on the core (common to all) SNPs, while also generating the default output files described above that are always generated.

#### Step 4c: Additional Tree Estimation Methods

The option -NJ will also estimate a tree based on the NJ method, while -ML will also estimate a tree based on the ML method. The command line is kSNP4 -in EEvirus.in -outdir EEV_Run3 -k 11 -core -NJ -ML |tee Run3Log.txt. These additions increased the run time from 7 to 8 s, and the number of output files was increased to 39. The added files include tree.SNPs_all.ML.tre and tree.SNPs_all.NJ.tre (the ML and NJ trees based on all SNPs) and tree.core_SNPs.ML.tre and tree.core_SNPs.NJ.tre (the ML and NJ trees based on the 67 core SNPs).

#### Step 4d: Simple Run With Annotation

Annotation provides information about each SNP that occurs in an annotated region of a genome and can be quite valuable for understanding the impact of an SNP. Annotation requires an annotation file that lists the genome sequences from which annotations can be taken. If you want the SNPs to be annotated, the data set must include some annotated genomes.

##### Step 4d1: Make a List of the Annotated Genomes

The easiest way to list the annotated genomes is to extract the genome names from the input file: type genomeNames4 infileName.in annotatedGenomes, and for this example, type genomeNames4 EEvirus.in annotatedGenomes.

The output is a file named annotatedGenomes (the second argument to the command).

In this example, all of the genomes are annotated, so the annotatedGenomes file lists all of the genomes:

EEE_PE-0_0155

EEE_FL93-939

EEE_Georgia97

EEE_ref_gi21218484

EEE_Florida91-4697

EEE_NJ-60

EEE_BeAr436087

EEE_NorthAmerican_antigenic_variety

EEE_PE-3_0815

EEE_PE6

Always edit the annotation file to remove names of any unannotated genomes.

##### Step 4d2: Run With Annotations

Be sure that Terminal is in the directory that contains both the input file and the annotatedGenomes file and enter kSNP4 -in EEvirus.in -outdir EEV_Run4 -k 11 -annotate annotatedGenomes |tee Run4Log.txt. The -annotate option says “annotate the SNPs based on the genomes listed in the file annotatedGenomes.”

Run 4 that sets the output directory to EEV_Run4 is identical to Run 1, except that it includes annotation. Annotation increased the time from 7 to 44 s and added 4 new output files. Two of these are: headers.annotate_list (a list of the headers of the fasta files used for annotation) and genbank_from_NCBI.gbk (the concatenation of all of the GenBank annotations that were downloaded). Both can be ignored.

The annotation_summary file provides information about each of the SNP locus as a table that shows for each SNP locus: the alternative nucleotides, alternative codons, whether the SNP is synonymous or nonsynonymous, and the gene product.

The SNPs_all_annotated file provides more detailed information about each SNP. For instance, in the EEvirus data set, SNP numbered 0 is present in only 2 genomes, while SNPs 5 and 6 are present in 8 genomes. For each genome in which the SNP is present, the file shows the genome ID, the accession number, the position of the SNP in that genome, the codon, the amino acid encoded, the peptide surrounding the SNP locus, and the gene product.

A comparison of the SNPs_all files from Run 1 and Run 4 shows that the Run 4 version includes the position of the SNP in the genome and whether it is on the forward or reverse strand.

## Annotation Strategy

In this example, annotation did not increase the run time to a great extent, but this is attributed to the fact that the data set consisted of only 10 small viral genomes, all of which were used for annotation. When dealing with larger genomes such as bacterial or yeast genomes, and more annotated genomes, the run time can increase dramatically compared with a run without annotation.

Often, the purpose of a kSNP4 analysis is to determine the relationships of a set of laboratory genomes to each other and perhaps to other known genomes. If a set of known genomes is included, it is helpful to ensure that these are annotated genomes. The first step would be to do a run without annotation and to print a drawing of the resulting phylogeny. This enables one to see the various clusters of the laboratory genomes and their relationships with the annotated genomes. One can then identify which of the annotated genomes are closely related to the target laboratory genomes. For the sake of efficiency and time, only those closely related genomes should be included on the annotatedGenomes list when the run is repeated with annotation.

## Results Storage Matters

kSNP4 writes a *lot* of files during its process, and these can consume a considerable amount of temporary space on your drive. For instance, on a data set of 400 *E. coli* genomes, kSNP4 used a peak of 142 GB of hard drive storage! At the end of the run, it deleted all but 20 files, and the resulting output directory contained 8.8 GB.

If you are using very large data sets, be sure that you have sufficient free space to accommodate the files that are written during the run.

## Drawing Trees With MEGA

Download MEGA for your operating system https://www.megasoftware.net/. Double-click the MEGA installer.pkg and follow the on-screen instructions to install it.

MEGA is a typical app with windows, menus, etc. Start MEGA, and from the File menu, choose Open a File/Session, and then navigate to the folder containing the .tre file(s), e.g. tree.parsimony.tre. MEGA will open the Tree Explorer window showing the tree. The Tree Explorer window allows you to manipulate the drawing including changing the format, re-rooting the tree, choosing whether or not to display clade confidence statistics, etc. “Building phylogenetic trees from molecular data with MEGA” (Hall 2013) provides details of using MEGA. Although it discusses an earlier version of MEGA, MEGA5, the tree-drawing features are unchanged.

Do read about rooted versus unrooted trees in that paper and about rooting trees with an out group. The outgroup genome should be that of a closely related species of the same genus.

## Supplementary Material

msad235_Supplementary_Data
