# Supplementary figures and images for: Building Phylogenetic Trees From Genome Sequences With kSNP4
Source: Mol Biol Evol. 2023 Nov 9;40(11):msad235. doi: 10.1093/molbev/msad235 (PMC10640685; doi:10.1093/molbev/msad235)

Cubic fit

Hours

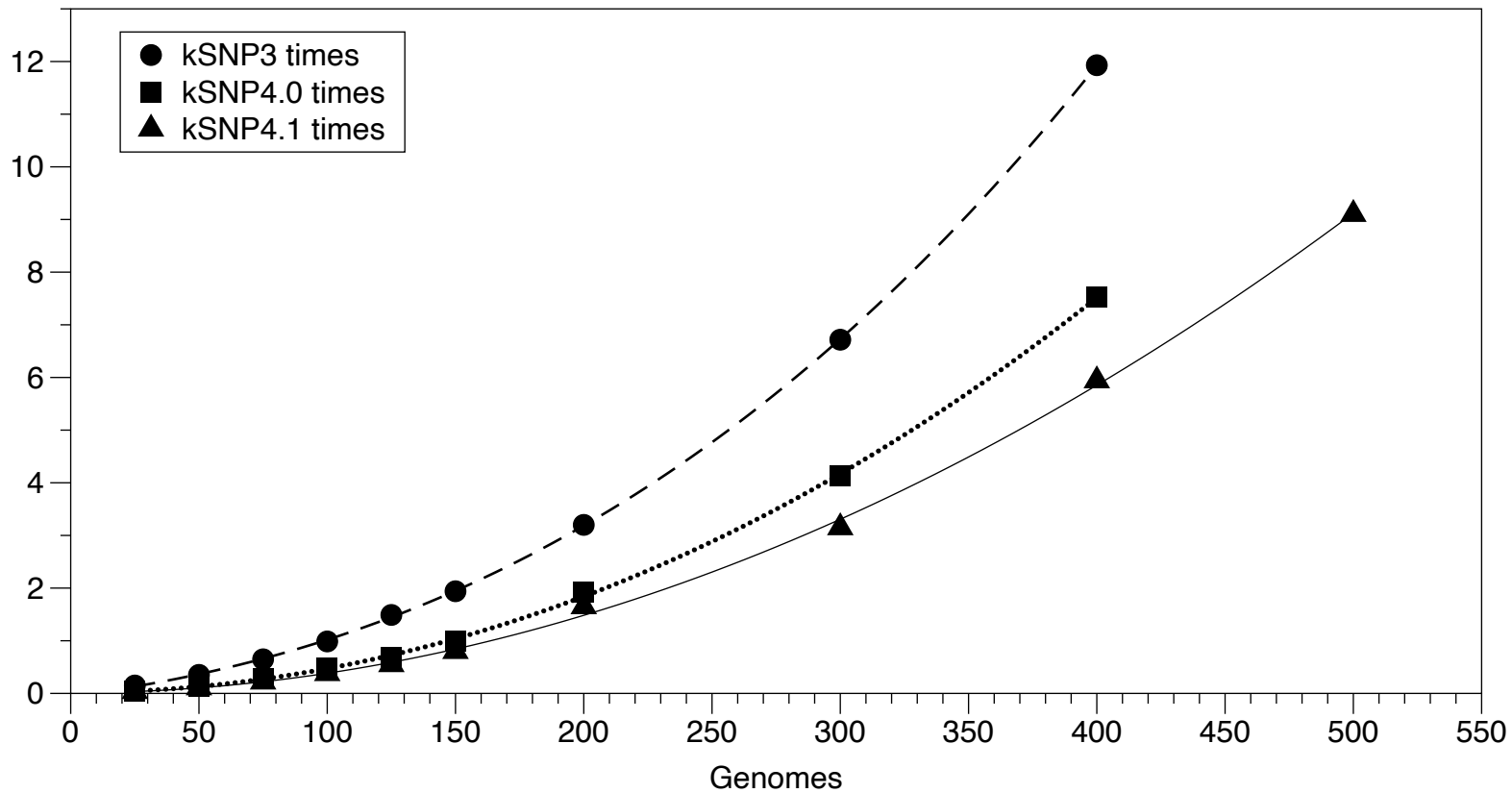

Supplement: msad235_Supplementary_Data [file msad235_supplementary_data.pdf]
